# The role of different acoustic environmental stimuli on manual dexterity

**DOI:** 10.1371/journal.pone.0307550

**Published:** 2024-07-22

**Authors:** Paola Adamo, Anna Fassi, Federico Temporiti, Davide De Leo, Giorgia Marino, Raffaello Furlan, Franca Barbic, Roberto Gatti, Isabella Barajon

**Affiliations:** 1 Physiotherapy Unit, IRCCS Humanitas Research Hospital, Rozzano, Milan, Italy; 2 Department of Biomedical Sciences, Humanitas University, Pieve Emanuele, Milan, Italy; 3 Internal Medicine, IRCCS Humanitas Research Hospital, Rozzano, Milan, Italy; 4 IRCCS Humanitas Research Hospital, Rozzano, Milan, Italy; Opole University of Technology: Politechnika Opolska, POLAND

## Abstract

Music has been reported to facilitate motor performance. However, there is no data on the effects of different acoustic environmental stimuli on manual dexterity. The present observational study aimed at investigating the effects of background music and noise on a manual dexterity task in young, middle-aged and elderly subjects. Sixty healthy, right-handed subjects aged between 18 and 80 years were enrolled. Twenty young (mean age: 22±2 years), 20 middle-aged (mean age: 55±8 years) and 20 elderly (mean age: 72±5 years) subjects performed the Nine Hole Peg Test (NHPT) in four different acoustic environments: silence (noise < 20dBA), classical music at 60dBA, rock music at 70 dBA, and a noise stimulus at 80dBA. Performance was recorded using an optical motion capture system and retro-reflective markers (SMART DX, 400, BTS). Outcome measures included the total test time and peg-grasp, peg-transfer, peg-in-hole, hand-return, and removing phases times. Normalized jerk, mean and peak of velocity during transfer and return phases were also computed. No differences were found for NHPT phases and total times, normalized jerk, peak of velocity and mean velocity between four acoustic conditions (p>0.05). Between-group differences were found for NHPT total time, where young subjects revealed better performance than elderly (p˂0.001) and middle-aged (p˂0.001) groups. Music and noise stimuli in the considered range of intensity had no influence on the execution of a manual dexterity task in young, middle-aged and elderly subjects. These findings may have implications for working, sportive and rehabilitative activities.

## Introduction

Interest in how the environment impacts daily life and working activities has strongly increased over the last thirty years. Environmental factors are defined as ‘the physical, social and attitudinal environments in which people live and conduct their lives’ [1]. These factors include climate, light and sound, and may facilitate or inhibit activities and social participation, as described by the international classification of functioning, disability and health model [1].

When considering acoustic environments, literature data have reported that human behavior is influenced by sound characteristics, as in the case of noise or music [2–7]. Studies have shown that annoying sound exposure may cause alterations in psychological and physical states [2–7]. The World Health Organization has also underlined the effects of communication interference and noise annoyance on cardiovascular system, work performance, social behaviors, and hearing impairments [2]. Moreover, noise exposure may affect demanding cognitive tasks [4], and negatively influences visual functions, concentration, and attention [3], increasing the number of errors and work accidents [4]. Conversely, positive influence of background music at workplace is still controversial. While Shih et al. have reported improved work-attention performance associated with pleasant background music [5], other authors have described negative effects of music on concentration and attention during some work activities [7].

Interestingly, some studies have also demonstrated that noise during hospital stay (i.e., produced by medical devices, nursing activities, etc.) may increase healing time, analgesic use, and readmission rate [6]. On the other hand, music seems to facilitate recovery after surgery, reducing pain, postoperative stress, blood pressure, heart and respiratory rates [8].

Music has been demonstrated to enhance motor performance during sport such as running by inducing positive psychological responses, reducing perceived exertion and fatigue, and improving oxygen consumption [9]. Additionally, the incorporation of music into rehabilitative sessions may enhance neurorehabilitation motor outcomes through auditory feedback and rhythmic acoustic stimuli, particularly in the case of central nervous system disease [10,11]. According to these observations, neuroimaging studies have showed that music enhances the activation of brain regions of the motor system, such as the primary motor cortex, supplementary motor area, dorsal and ventral pre-motor areas [12].

A previous study has also demonstrated that positive and negative emotions induced by music may improve motor learning in terms of motor sequence learning, when compared to a neutral acoustic environment [13]. However, the specific characteristics of music on motor performance with regards to motor control have never been analyzed.

Furthermore, the association between music rhythm and movements has been investigated without considering music melody or listeners’ preferences [12]. Motor performance is the result of several factors, such as muscular strength, balance, coordination and dexterity. Nevertheless, how music influences these factors has never been explored.

To the best of our knowledge, no studies have investigated the effects of different acoustic environmental stimuli compared to a silent condition on manual dexterity. Manual dexterity represents an indicator of motor control during hand and fingers movements [14]. When considering the importance of manual dexterity during daily, sportive activities and in rehabilitation field, this information may assume relevance, especially in challenging conditions characterizing several daily life activities [15,16].

Worsening in motor control and sensorimotor integration has been described in older adults compared to young healthy subjects [17]. Since differences in multisensory integration, defined as the processing of multisensory stimuli through which information from multiple sensory modalities are combined, have been reported between young and elderly subjects, acoustic stimuli may have a different effect on manual dexterity of adults differently across various age groups [18].

These findings could increase knowledge on the role of acoustic stimuli in a specific motor performance and on the usefulness of a specific acoustic enriched environment.

The study aim was to investigate the effects of different acoustic environmental stimuli such as silence, classical music, rock music, and noise on manual dexterity task in young, middle-aged and elderly subjects. The hypothesis is that a specific acoustic environment may influence motor task performance, either enhancing or disrupting it depending on the elicited emotional response.

## Material and methods

### Participants

Sixty healthy subjects with right-hand dominance were enrolled from October to December 2023 and divided into 3 groups according to age criteria: 1) Young group, ranging from 18 to 35 years (YG, n = 20), 2) Middle-age group, ranging from 35 to 64 years (MG, n = 20), 3) Elderly group, ranging from 65 to 80 years (EG, n = 20). The right upper-limb dominance was defined using a Edinburgh Handedness Inventory score higher than 40 (Oldfield, 1971) [19]. Exclusion criteria were auditory deficits or orthopedic and/or neurological disorders affecting the dominant upper limb. Music experts like musicians or dancers were also excluded. Participants signed a written informed consent form, and the study was approved by the Ethical Committee for Human Investigation of Humanitas Research Hospital (protocol number: CLF21/01).

### Experimental protocol

The instrumental Nine Hole Peg Test (NHPT) was used to assess manual dexterity [20]. Participants were seated on an armless height-adjustable chair with hip and knee flexed at 90°, with the NHPT board positioned in front of them on an adjustable height table. The NHPT consists of grasping nine pegs from a container one-by-one and placing them into nine holes. After having inserted the nine holes, subjects were asked to remove each peg back into the container. The test was completed only with the dominant upper limb as quickly as possible. During NHPT performance, four different auditory environmental stimuli were administered through earphones (Apple Airpods): 1) *Silence*: noise-free environment (Noise < 20 dBA); 2) *Noise*: noisy environment consisting of a soundtrack with household noise, including sounds of pots, dishes, and drilling machine (≈ 80 dBA); 3) *Classical music*: Beethoven’s ‘Moonlight Sonata’ soundtrack (≈ 60 dBA); 4) *Rock music*: Jet’s song “Are you gonna be my girl” (≈ 70 dBA). For the rock music condition, we pre-recorded a 40-second track, avoiding any vocal content and recording exclusively the rhythmic component. The intensity of acoustic stimuli was quantified by *Sonic Visualizer* software. The noise intensity of 80 dBA corresponds to an intense city traffic condition, and it is widely described as annoying in terms of concentration [21].

Emotional responses evoked by environmental auditory stimuli were also investigated after each trial. Participants had to attribute a score from 1 (strongly disagree) to 7 (strongly agree) to four adjectives (*joyful*, *serene*, *annoyed* and *disturbed*) to describe emotional responses to each stimulus [22,23]. All the subjects executed the task once in each auditory environmental in order to minimize practice effect [24]. The different acoustic environments were administered in a randomized computer-generated order and a five-minute period of rest followed each trial.

### Data collection and processing

Kinematics during NHPT executed with the right hand were collected through an optoelectronic system (SMART-DX, BTS, Italy), consisting of 8 cameras sampling at 100 Hz. Eight spherical (10 mm diameter) retro-reflecting markers were placed bilaterally on acromion, on the jugular incisura, on the right lateral epicondyle of the humerus, radial and ulnar styloid processes, between the head of the second and the third metacarpal bone, and middle phalanx of the index finger. Three additional markers were placed on the edges of the table to define the global reference system. Before administering the test, subjects performed a 10-second static test for the calibration where 4 additional markers were placed on the NHPT board.

NHPT performance was segmented into 4 different phases for every single attempt: peg-grasp, peg-transfer, peg-in-hole and hand-return phases. Moreover, the final removing phase, which consists of the phase in which participants were asked to remove the pegs and replace them back into the container, was also analysed.

Raw marker data were filtered using a fourth-order low-pass Butterworth filter (cut-off 4 Hz). Subsequently, the following kinematic indexes were computed during NHPT: total and single phases time (peg-grasp, peg-transfer, peg-in-hole, hand-return), normalized jerk, mean and peak velocity during peg transfer and hand-return phase [20]. Subjects performed one trial for each environmental stimulus.

Mean and Peak velocity consisted of the average and the maximum value of velocity computed from the finger index marker, whereas Normalized jerk (N-Jerk) was used to measure movement smoothness. N-Jerk was computed as the time integral of squared jerk and divided by *length*^*2*^*/duration*^*5*^ in order to remove the influence of distance covered by the index finger and time of execution, as described in previous studies [25,26].

### Statistical analysis

The normality distribution of the variables was checked by the Kolmogorov-Smirnov test. Data were expressed as mean and standard deviation, while Chi-Square test was used to assess between-group differences for gender distribution.

Two-way repeated-measures ANOVA with gender as covariate was used to compare kinematic variables in the four environmental conditions. Bonferroni post-hoc tests were used for pairwise comparisons.

The scores obtained from *joyful* and *serene*, and from *disturbed* and *annoyed* were averaged and used to quantify positive and negative responses toward environmental stimulus, respectively.

Two-way repeated-measures ANOVA (within-subject factors: emotional responses [2 levels: positive valence; negative valence]; sound environment [4 levels: silence, classical music, rock music, noise]) was used to compare emotional responses in different environmental conditions. In case of significant interactions or main effects, Bonferroni post-hoc tests were used.

Pearson’s correlation coefficients were calculated to evaluate any association between emotional responses and kinematic variables for each environmental condition. P-values were Bonferroni corrected by multiplying by 8 to account for multiple comparisons.

Statistical level of significance was set at α = 0.05 and statistical analysis was performed using SPSS version 25.0 for Windows.

## Results

Twenty young (mean age 22±2 years; mean height 177.5±6.8 cm; mean weight 74.5±14.3 kg; 15 males and 5 females), 20 middle-aged (mean age 55±8; mean height 163.4±7.3 cm; mean weight 66.3±16.8 kg; 2 males and 18 females), and 20 elderly subjects, (mean age 72±5; mean height 169.7±8.7 cm; mean weight 74±14.3 kg; 11 males and 9 females) completed the recording sessions correctly and no dropouts occurred. Differences in the proportion of males and females were found between the three groups (p˂0.001), specifically higher proportion of females was found in MG respect to YG (p˂0.001) and EG (p = 0.002).

### Effects of different auditory environments on motor task

**Fig 1:**
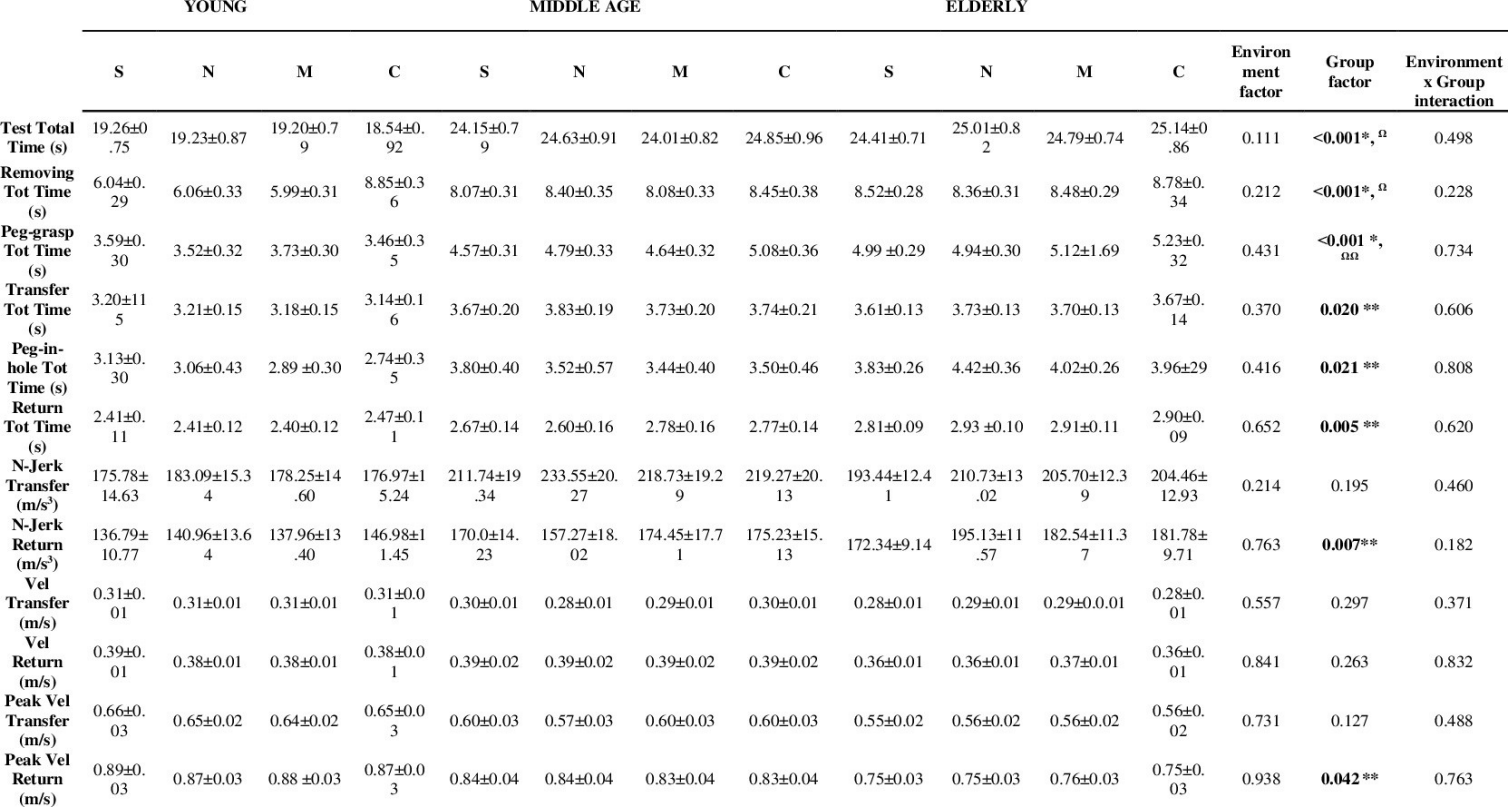
Comparison of kinematic indices between silence, classical music, rock music and noise stimuli (repeated measures ANOVA). Data are shown as mean and standard deviation. S: Silence, N: Noise, M: Rock music, C: Classical music. * Young vs Elderly: p˂0.001; ** Young vs Elderly: p˂0.05; **Ω** Young vs Middle-age: p˂0.001; **ΩΩ** Young vs Middle-age: p˂0.05.

Kinematics indexes in the four auditory environments during NHPT performance are shown in **Fig 1**. No differences in terms of recorded parameters were found during noise, classical and rock music stimuli compared to silence condition in both YG, MG and EG. In particular, music and noise did not influence the N-Jerk, mean and peak velocity and total and subphases execution times of the NHPT. Between-group post-hoc analysis revealed better NHPT total time in YG compared to EG (p˂0.001) and MG (p˂0.001) groups.

Specifically, YG revealed significant differences compared to EG in NHPT phases requiring peg manipulation, such as Peg-grasp time (p˂0.001) and Peg-in-hole time (p = 0.008). Moreover, significant differences were found for Transfer, Return and Removing phases. In particular, YG reached higher Peak Velocity during Return phase (p = 0.006), completing the Return phase in less time (p = 0.002) and through smoother movements expressed by N-Jerk (p = 0.008) compared to EG. Finally, lower Removing and Transfer time were found in YG compared to EG (p˂0.001 and p = 0.025, respectively).

Differences in NHPT total time between YG and MG mainly depend on lower Removing time in YG (p˂0.001) and Peg Grip time (p = 0.015).

No statistically significant differences were found between MG and EG in terms of subphases duration, peak and mean velocity and N-Jerk.

**Fig 2:**
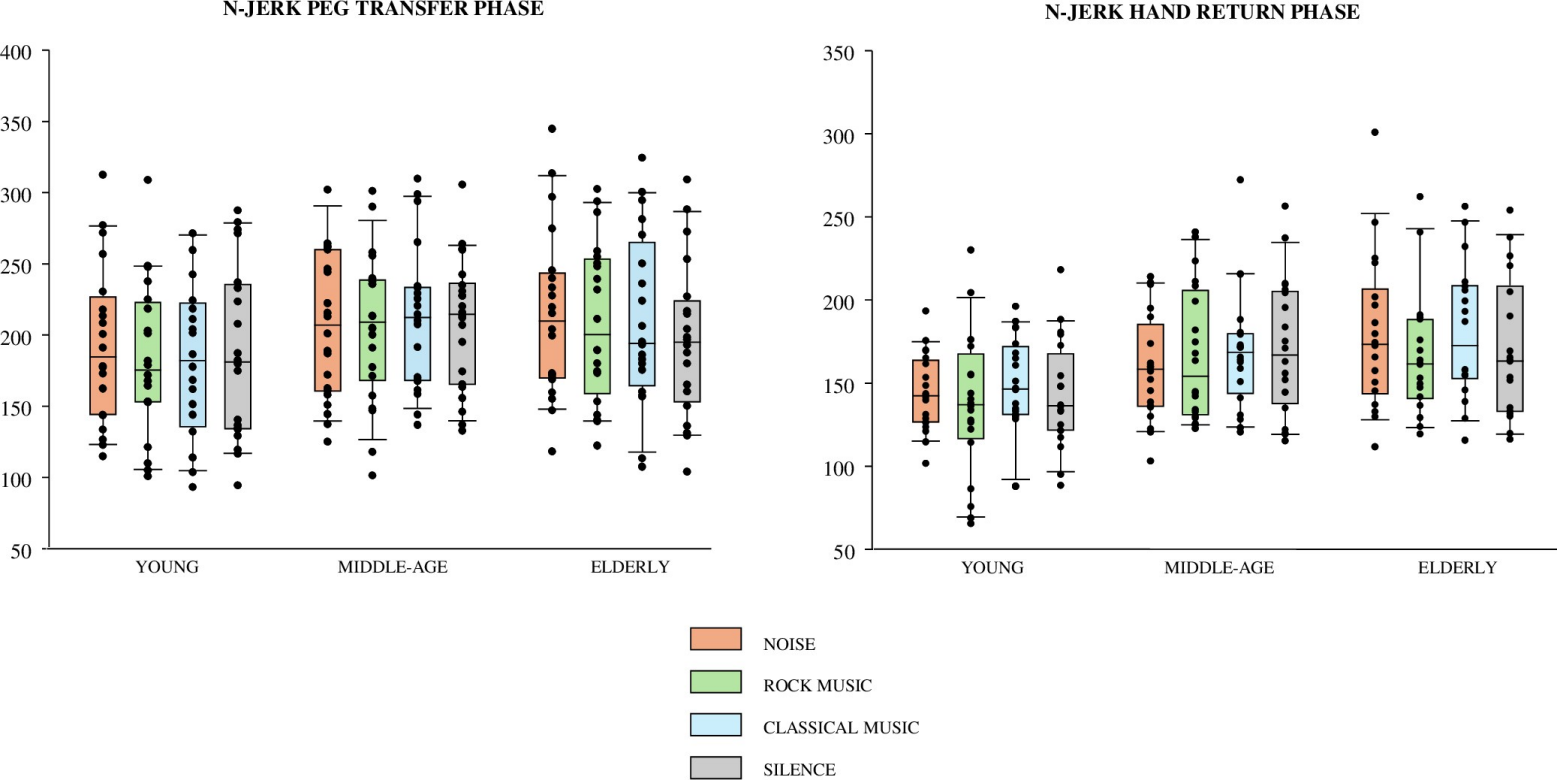
Normalized jerk during peg transfer and hand return phases in young, middle-age and elderly groups for each acoustic stimulus. Boxes represent the range between the first and the third quartile, the middle horizontal line is the median value, and the end of the vertical line are the maximum and minimum values (outliers are also shown). Dots represent the raw data of each participant in the different environmental conditions.

**Fig 2** represents raw data for N-jerk during transfer and return phases for study participants.

### Emotional response

The association between emotional responses and auditory stimuli is shown in **Table 1**. Silence, rock and classical music stimuli induced higher positive emotional values compared to noise, whereas noise stimulus induced higher negative emotional values compared to silence and music stimuli. Moreover, classical music stimulus resulted in higher positive score compared to silence condition.

**Table 1:** Comparison of emotional response in different environmental stimuli (two-way repeated-measures ANOVA, within-subject factors: Emotional Response [2 levels: Positive; negative]; Sound environment [4 levels: Silence, rock, classical, noise]).

	SILENCE	ROCK	CLASSICAL	NOISE	p-value
**Positive emotional response**	4.5 ± 1.3	5.1 ± 1.4	5.8 ± 1.1	2.1 ± 1.6	< 0.001*
**Negative emotional response**	1.7 ± 1.1	1.8 ± 1.2	1.2 ± 0.6	5.2 ± 1.8	< 0.001#
**p-value**	< 0.001	< 0.001	< 0.001	< 0.001	

* = noise VS rock p˂0.001; silence VS noise p < 0.001; classical VS noise p < 0.001; classical VS silence: p< 0.001.

# = silence VS noise p < 0.001; rock VS noise p < 0.001; classical VS noise p < 0.001.

Data are shown as mean ± standard deviation.

However, no correlations between emotional responses and kinematic parameters were found, except to low correlation between positive emotional response and some kinematics parameters of NHPT during classical music condition, as showed in **Table 2**.

**Table 2:** Correlation between emotional responses and kinematic parameters in different environmental stimuli (Pearson correlation coefficient and adjusted p-value).

	Silence		Noise		Rock Music		Classical Music	
**Emotional Response**	**+** **R(p)**	**-** **R(p)**	**+** **R(p)**	**-** **R(p)**	**+** **R(p)**	**-** **R(p)**	**+** **R(p)**	**-** **R(p)**
**Test Total Time (s)**	0.105 (p = 0.999)	- 0.1116 (p = 0.999)	0.150 (p = 0.999)	-0.099 (p = 0.999)	0.201 (p = 0.992)	- 0.173 (p = 0.999)	**0.361 (p = 0.04)**	- 0.265 (p = 0.32)
**Removing Time (s)**	0.076 (p = 0.999)	- 0.192 (p = 0.999)	0.153 (p = 0.999)	-0.157 (p = 0.999)	0.196 (p = 0.999)	- 0.248 (p = 0.448)	**0.387 (p = 0.016)**	-0.322 (p = 0.096)
**Peg-grasp (s)**	0.077 (p = 0.999)	- 0.105 (p = 0.999)	0.179 (p = 0.999)	- 0.089 (p = 0.999)	0.073 (p = 0.999)	0.082 (p = 0.999)	0.259 (p = 0.36)	-0.137 (p = 0.999)
**Peg-Transfer (s)**	0.223 (p = 0.696)	- 0.171 (p = 0.999)	0.004 (p = 0.999)	- 0.008 (p = 0.999)	0.083 (p = 0.999)	- 0.180 (p = 0.999)	0.183 (p = 0.999)	-0.165 (p = 0.999)
**Peg-in-hole (s)**	- 0.047 (p = 0.999)	0.114 (p = 0.999)	0.108 (p = 0.999)	- 0.021 (p = 0.999)	0.313 (p = 0.12)	- 0.223 (p = 0.688)	0.272 (p = 0.28)	- 0.224 (p = 0.68)
**Hand-return (s)**	0.109 (p = 0.999)	- 0.083 (p = 0.999)	0.062 (p = 0.999)	- 0.026 (p = 0.999)	0.057 (p = 0.999)	- 0.107 (p = 0.999)	0.243 (p = 0.488)	-0.119 (p = 0.999)
**Mean Velocity Transfer (m/s)**	- 0.302 (p = 0.152)	0.173 (p = 0.999)	0.016 (p = 0.999)	- 0.144 (p = 0.999)	- 0.016 (p = 0.999)	0.012 (p = 0.999)	-0.308 (p = 0.136)	0.145 (p = 0.999)
**Mean Velocity Return (m/s)**	- 0.199 (p = 0.999)	0.174 (p = 0.999)	- 0.035 (p = 0.999)	- 0.075 (p = 0.999)	0.143 (p = 0.999)	-0.085 (p = 0.999)	-0.245 (p = 0.472)	0.033 (p = 0.999)
**Peak Velocity Transfer (m/s)**	- 0.263 (p = 0.336)	0.106 (p = 0.999)	0.074 (p = 0.999)	- 0.164 (p = 0.999)	- 0.122 (p = 0.999)	0.153 (p = 0.999)	**-0.362 (p = 0.032)**	0.279 (p = 0.248)
**Peak Velocity Return (m/s)**	- 0.156 (p = 0.999)	0.054 (p = 0.999)	- 0.054 (p = 0.999)	- 0.016 (p = 0.999)	0.070 (p = 0.999)	- 0.085 (p = 0.999)	-0.340 (p = 0.064)	0.131 (p = 0.999)
**N-Jerk Transfer**	0.189 (p = 0.999)	- 0.157 (p = 0.999)	- 0.019 (p = 0.999)	- 0.013 (p = 0.999)	0.012 (p = 0.999)	- 0.160 (p = 0.999)	0.099 (p = 0.999)	-0.105 (p = 0.999)
**N-Jerk Return**	0.062 (p = 0.999)	-0.007 (p = 0.999)	0.099 (p = 0.999)	- 0.048 (p = 0.999)	-0.346 (p = 0.056)	- 0.033 (p = 0.999)	0.200 (p = 0.999)	-0.116 (p = 0.999)

**+**: Positive emotional response; **-**: Negative emotional response.

## Discussion

The study aim was to investigate the influence of different auditory stimuli on manual dexterity in young, middle-aged and elderly subjects. We investigated different age groups since we speculated that environmental stimuli may have different impacts on subjects of different ages. For example, elderly have been described to have lower ability in weighing relevant and irrelevant sensory information from the environment [18]. Therefore, it was reasonable to hypothesize that potentially distracting stimuli might have induced different effects on motor performance in older individuals, especially compared to younger subjects [18]. The decision to explore two different music stimuli raised from the recognition that identical music may generate very different emotional responses in different subjects [27]. A close interaction between brain auditory and motor areas has been described by neuroimaging studies [28], and the recruitment of motor regions has been demonstrated during music background listening [12]. However, in the current study, environmental sound stimuli (silence, rock and classical music and noise) revealed no influence on the kinematics of a manual dexterity task in healthy subjects. This finding was observed despite the positive emotional responses elicited by silence and music stimuli unlike the sound of noise which elicited opposite responses.

The lack of kinematic differences during the four sound stimuli may be attributed to a ‘sensory gating’ phenomenon, in which the central nervous system filters sensory information in order to prevent overstimulation of cortical areas [29]. In fact, inhibition of unnecessary environmental stimuli is essential to exclude irrelevant information and provide a correct task execution [30].

The emotional state elicited during noise or music stimuli was not associated with changes in kinematic parameters since only positive response during classical music stimulus reported a low positive correlation with the time of execution of NHPT and the Removing time. A low negative correlation with the peak of velocity during transfer phase was also observed in the classical music condition. Our findings suggested that healthy subjects are capable of filtering out useless stimuli and managing emotions they evoked, especially stimuli with a negative valence such as those occurring in a noisy environment.

Swaminathan and Schellenberg (2015) have reported the opportunity to elicit specific emotional states using sound associated with memories of positive or negative events [31]. In our study, participants associated noise with negative emotions, while they associated rock music, classical music, and silence with positive emotions. However, these associations were made within a context unlikely to have elicited specific emotional states. The current results revealed differences between young and elderly subjects in NHPT total time and in all NHPT phases, in agreement with previous data describing a worsening in motor control with aging [17].

Previous studies have demonstrated manual dexterity differences considering age and sex of participants [32,33]. However, these studies commonly assess only the total time taken to complete a manual dexterity test, without differentiating between different phases such as reaching, peg-manipulation and peg-moving phases, and without information regarding movement velocity and smoothness [33]. Based on the study results, instrumental assessment of NHPT provides insights for detecting differences, particularly between young and elderly subjects. Specifically, young subjects revealed better performance compared to elderly subjects in peg manipulation tasks, which required higher manual dexterity [17], but also in Removing and Return phase, in which young subjects executed upper limb movements faster and smoother respect to elderly group. Normalized jerk has been commonly used as a measure of movement trajectory smoothness and has been shown to be greater in elderly adults during goal-directed movements [34]. Only Removing time and Peg-grasp time discriminated between young and middle-aged healthy subjects, who performed these sub-phases slower. On the other hand, no differences in NHPT total time, sub-phases duration or movement smoothness were found in NHPT between middle-aged and elderly subjects.

Despite the differences between subjects of different ages, young and older subjects executed the motor task without the influence of a noisy or musical environment.

The study of potential effects of sound environments on motor performance may play an important role when considering working environments, since noise is considered as one of the most dangerous workplace exposures [35]. Several studies have reported hearing impairments and cardiovascular disorders as a consequence of a long-term noise exposure [2]. Furthermore, noise exposure may adversely affect cognitive task performance, increasing the number of errors [3]. To date, no studies have explored the noise effects on manual dexterity. Although in our study music and noise exposition does not influence manual dexterity, it is worth noting that our experimental setting does not simulate working characteristics, and it cannot be excluded that results could be different for longer duration of noise exposures.

Some limitations of this study need to be underlined. First, we cannot generalize our findings to all types of music stimuli. In fact, different types of music, including different rhythmic contents and melodies potentially more aligned with participants’ preferences might have led to different results in terms of motor performance. A second limitation was the lack of neurophysiological measurements or neuroimaging data to support the current findings. In fact, the effects of different enriched environments including auditory stimuli might modify brain activity in order to control potential distractors during execution of the same task.

## Conclusions

In conclusion, acoustic environmental stimuli of silence, music and noise had no influence on motor execution and coordination of a manual dexterity task in young, middle-aged, and elderly subjects.

## Supporting information

S1 Checklist STROBE statement—checklist of items that should be included in reports of observational studies. (DOCX)

S1 Dataset (XLSX)
