# Supplementary material for: The role of different acoustic environmental stimuli on manual dexterity
Source: PLoS One. 2024 Jul 22;19(7):e0307550. doi: 10.1371/journal.pone.0307550 (PMC11262680; doi:10.1371/journal.pone.0307550)
Supplement: S1 Checklist — (DOCX) [file pone.0307550.s001.docx]

STROBE Statement—checklist of items that should be included in reports of observational studies

|  | Item No. | Recommendation | Page  No. | Relevant text from manuscript |
| --- | --- | --- | --- | --- |
| **Title and abstract** | 1 | (*a*) Indicate the study’s design with a commonly used term in the title or the abstract | 2 (abstract) | The present observational study was aimed at investigating the upper limb coordination during simple and demanding motor tasks performed with background music or noise |
|  |  | (*b*) Provide in the abstract an informative and balanced summary of what  was done and what was found | 2 (abstract) | The present study was aimed at investigating the upper limb coordination during simple and demanding motor tasks performed with background music or noise. The same motor task performed in a silent environment was considered as a reference condition. Twenty-five right-handed subjects (mean age 24±3 years) were asked to move a spoon containing a sponge ball with the right upper limb in the three different acoustic environments: silence (i.e. noise < 20dBA), classic music at 45dBA, and during a noise stimulus at 70dBA. The task was executed in a simple and challenging motor-cognitive dual-task condition. Performance was recorded using an optical motion capture system, and normalized jerk, peak of velocity, mean velocity and execution time were analyzed. Surface electromyography of flexor and extensor carpi radialis, biceps brachii and triceps brachii muscles was also expressed as root mean square (RMS).  No differences were found for normalized jerk, peak of velocity, mean velocity, execution time and RMS between the three acoustic stimuli during simple and challenging conditions (p>0.05). |
| Introduction | | | |  |
| Background/rationale | 2 | Explain the scientific background and rationale for the investigation being reported | 2 - 3 | Recently, the effects of noise in hospital environment on patients’ recovery have been largely studied. Some studies suggested that irregular noise during hospital stay (i.e., produced by medical devices, nursing activities, etc.) may increase healing time, analgesic use, and readmission rate.^6^ Moreover, chronic noise exposure increases stress hormones production and release, blood pressure values and the risk of developing cardiovascular disorders.^8^ On the other hand, music seems to facilitate recovery after surgery, reducing pain and post-surgery stress. In addition, blood pressure, heart and respiratory rates seem to be reduced by a pleasant music during hospital stay.^8^  Previous studies reported that the use of music in rehabilitation sessions may have positive effects on emotional state, movement stimulation, compliance, and enjoyment.^9^ In particular, motor practice combined with music stimulation, such as the use of auditory feedback during motor task performance, revealed benefits in post-stroke motor rehabilitation, enhancing neurorehabilitation motor outcomes.^10^ Moreover, rhythmic acoustic stimuli improved motor function and walking in people with Parkinson’s disease, epilepsy, and multiple sclerosis.^11^ According with these observations, neuroimaging studies showed that music enhances the activation of brain regions of the motor system, such as the primary motor cortex, supplementary motor area, dorsal and ventral pre-motor areas.^12^  Nevertheless, the type of music characteristics able to influence motor performance have never been analyzed. Several studies associated music rhythm and movements, without considering music melody or listeners’ preferences .^12^ Classic music is often used as background in rehabilitative enriched environments although its rhythmic component is not predominant.^13^ Moreover, motor performance is determined by several factors, such as muscular strength, balance, coordination and dexterity, but what of the aforementioned factors are influenced by music has never been defined.  To the best of our knowledge, no studies have investigated the effects of different acoustic stimuli (noise and classic music) compared to silence on upper limb coordination during task-oriented movements. When considering importance of upper limb coordination in rehabilitation and during daily and sportive activities, this information may assume relevance, especially in challenging condition characterizing several daily life activities, such as dual task performance.^14,15^ |
| Objectives | 3 | State specific objectives, including any prespecified hypotheses | 3 | When considering importance of upper limb coordination in rehabilitation and during daily and sportive activities, this information may assume relevance, especially in challenging condition characterizing several daily life activities, such as dual task performance.^14,15^ These findings could increase knowledge on the role of acoustic stimuli in a specific motor performance and on the usefulness of a specific acoustic enriched environment.  The study aim was to examine upper limb coordination during single-task and dual-task performance, executed in three different sound environments: silence, classic music, and noise. |
| Methods | | | |  |
| Study design | 4 | Present key elements of study design early in the paper | 4-5 | **Participants**  Twenty-five healthy right-handed subjects (13 men and 12 women; age 24.4 ± 3.5 years, range 20-30 years; mean height 171 ± 9.6 cm; mean weight 67.2 ± 11.4 kg) were enrolled from students and employees of Humanitas Research Hospital from January to March 2022. The right upper-limb dominance of the enrolled subjects was defined by the Edinburgh Handedness Inventory score higher than 40 (Oldfield, 1971).^16^ Exclusion criteria were the presence of auditory deficits or orthopedic and/or neurological disorders affecting the dominant upper limb. Music experts like musicians or dancers will be also excluded. Participants signed a written informed consent form, and the study was approved by the Ethical Committee for Human Investigation of Humanitas Research Hospital (protocol number: CLF21/01).  **Experimental protocol**  The participants of the study were seated on an armless height adjustable chair with hip and knee flexed at 90°, feet on the ground and face oriented forward. Upper limbs were positioned with hand palms on a table placed in front of them, arms closed to the chest and elbows flexed at 90°. A spoon containing a sponge ball (weight: 90 grams; diameter: 7 cm) was placed on the table at body midline and 90% of the dominant upper arm length.^17^  All the subjects wore ad hoc earphones in order to receive different acoustic stimuli.  All subjects were asked to perform a reaching-grasping-transporting task, moving the spoon with the dominant upper-limb, without dropping the ball.  The task described was performed in a *simple condition* and in a *challenging condition*. In particular, the simple condition consisted of task performance alone, whereas in the challenging condition participants had to count aloud backwards by three starting from a number between 150 and 450, simultaneously with the execution of the task.^19,20^  During both simple and challenging condition performance, three different auditory environmental stimuli were administered through earphones (TAOTRONICS SoudLiberty 53).  The different acoustic environments were administered in a randomized computer-generated order. |
| Setting | 5 | Describe the setting, locations, and relevant dates, including periods of recruitment, exposure, follow-up, and data collection | 4 | Twenty-five healthy right-handed subjects (13 men and 12 women; age 24.4 ± 3.5 years, range 20-30 years; mean height 171 ± 9.6 cm; mean weight 67.2 ± 11.4 kg) were enrolled from students and employees of Humanitas Research Hospital from January to March 2022. |
| Participants | 6 | (*a*) *Cohort study*—Give the eligibility criteria, and the sources and methods of selection of participants. Describe methods of follow-up  *Case-control study*—Give the eligibility criteria, and the sources and methods of case ascertainment and control selection. Give the rationale for the choice of cases and controls  *Cross-sectional study*—Give the eligibility criteria, and the sources and methods of selection of participants | 4 | Healthy right-handed subjects (13 men and 12 women; age 24.4 ± 3.5 years, range 20-30 years; mean height 171 ± 9.6 cm; mean weight 67.2 ± 11.4 kg) were enrolled. The right upper-limb dominance of the enrolled subjects was defined by the Edinburgh Handedness Inventory score higher than 40 (Oldfield, 1971).^16^ Exclusion criteria were the presence of auditory deficits or orthopedic and/or neurological disorders affecting the dominant upper limb. Music experts like musicians or dancers will be also excluded. |
|  |  | (*b*) *Cohort study*—For matched studies, give matching criteria and number of exposed and unexposed  *Case-control study*—For matched studies, give matching criteria and the number of controls per case |  | NA |
| Variables | 7 | Clearly define all outcomes, exposures, predictors, potential confounders, and effect modifiers. Give diagnostic criteria, if applicable | 5 | During both simple and challenging condition performance, three different auditory environmental stimuli were administered through earphones (TAOTRONICS SoudLiberty 53): 1) *Silence:* noise-free environment (Noise < 20 dBA); 2) *Noise*: noisy environment consisting of a soundtrack with household noise, including sounds of pots, dishes, and drilling machine (≈ 70 dBA); 3) *Music*: Beethoven’s ‘Moonlight Sonata’ soundtrack (≈ 45 dBA).  Kinematic were recorded by an optical motion capture system (BTS SMART-DX, BTS, Italy) with 8 infrared cameras sampling at 100 Hz.  The following parameters were extracted during the task performance: normalized jerk (NJ), peak of velocity (PeakVel, m/s), mean velocity (MeanVel, m/s) and execution time (Time, sec).^24,27^ MeanVel and PeakVel were the average and the maximum value of velocity computed from the finger index marker, whereas NJ was used to measure movement smoothness. |
| Data sources/ measurement | 8* | For each variable of interest, give sources of data and details of methods of assessment (measurement). Describe comparability of assessment methods if there is more than one group | 5 | The following parameters were extracted during the task performance: normalized jerk (NJ), peak of velocity (PeakVel, m/s), mean velocity (MeanVel, m/s) and execution time (Time, sec).^24,27^ MeanVel and PeakVel were the average and the maximum value of velocity computed from the finger index marker, whereas NJ was used to measure movement smoothness. Normalized jerk (NJ) was computed as the time integral of squared jerk and dividing it by length^2^/duration^5^, in order to remove the influence of distance covered by index finger and time of execution, as described in previous studies.^27,28^  Neuromuscular activation was detected using surface electromyography (sEMG) (FREEEEMG 1000, BTS, Italy) applied according to SENIAM recommendations.^29^ |
| Bias | 9 | Describe any efforts to address potential sources of bias | 5 | The different acoustic environments were administered in a randomized computer-generated order. |
| Study size | 10 | Explain how the study size was arrived at |  | NA |

Continued on next page

| Quantitative variables | 11 | Explain how quantitative variables were handled in the analyses. If applicable, describe which groupings were chosen and why | 5-6 | Raw kinematics data were filtered using a fourth-order low-pass Butterworth filter (cut-off 4 Hz). The start and the end of the task were identified when the velocity of the index finger marker exceeded or dropped below 0.005 m/s.^25^ The first and the last repetitions of each task were removed, and data analysis was carried out for the second, third and fourth repetitions of each task executed during the administration of the same stimulus.^26^The following parameters were extracted during the task performance: normalized jerk (NJ), peak of velocity (PeakVel, m/s), mean velocity (MeanVel, m/s) and execution time (Time, sec).^24,27^  Raw sEMG signals were processed using a 20 Hz high-pass and a 450 Hz low-pass fourth order Butterworth filter. Subsequently, the root mean square (RMS) of the signal was computed for consecutive epochs of 300 milliseconds throughout the entire task.^32^ |
| --- | --- | --- | --- | --- |
| Statistical methods | 12 | (*a*) Describe all statistical methods, including those used to control for confounding | 6 | One-way repeated-measures ANOVA was used to compare kinematic and sEMG variables in Silence, Music and Noise conditions. Bonferroni post-hoc tests were used for pairwise comparisons.  Two-way repeated-measures ANOVA (within-subject factors: emotional responses [2 levels: positive valence; negative valence]; sound environment [3 levels: Silence, Music, Noise]) was used to compare emotional responses in different environmental conditions. In case of significant interactions or main effects, Bonferroni post-hoc tests were used.  Pearson’s correlation coefficients were calculated to evaluate any association between emotional responses and kinematic variables for each environmental condition.  Finally, paired t-test was used to compare *simple task* and *challenging task* within each environmental condition.  Statistical level of significance was set at α = 0.05 and statistical analysis was performed using SPSS version 25.0 for Windows. |
|  |  | (*b*) Describe any methods used to examine subgroups and interactions | 6 | Two-way repeated-measures ANOVA (within-subject factors: emotional responses [2 levels: positive valence; negative valence]; sound environment [3 levels: Silence, Music, Noise]) was used to compare emotional responses in different environmental conditions. In case of significant interactions or main effects, Bonferroni post-hoc tests were used. |
|  |  | (*c*) Explain how missing data were addressed |  | There was no missing data |
|  |  | (*d*) *Cohort study*—If applicable, explain how loss to follow-up was addressed  *Case-control study*—If applicable, explain how matching of cases and controls was addressed  *Cross-sectional study*—If applicable, describe analytical methods taking account of sampling strategy |  | NA |
|  |  | (*e*) Describe any sensitivity analyses |  | NA |
| Results | | | | |
| Participants | 13* | (a) Report numbers of individuals at each stage of study—eg numbers potentially eligible, examined for eligibility, confirmed eligible, included in the study, completing follow-up, and analysed | 7 | The 25 participants completed all the recording sessions correctly. No additional task repetition was needed due to ball dropping from the spoon. |
|  |  | (b) Give reasons for non-participation at each stage |  | NA |
|  |  | (c) Consider use of a flow diagram |  | NA |
| Descriptive data | 14* | (a) Give characteristics of study participants (eg demographic, clinical, social) and information on exposures and potential confounders | 4 | Twenty-five healthy right-handed subjects (13 men and 12 women; age 24.4 ± 3.5 years, range 20-30 years; mean height 171 ± 9.6 cm; mean weight 67.2 ± 11.4 kg) were enrolled |
|  |  | (b) Indicate number of participants with missing data for each variable of interest |  | No participants with missing data were present. |
|  |  | (c) *Cohort study*—Summarise follow-up time (eg, average and total amount) |  | No follow-up sessions were present (single evaluation session). |
| Outcome data | 15* | *Cohort study*—Report numbers of outcome events or summary measures over time |  | NA |
|  |  | *Case-control study—*Report numbers in each exposure category, or summary measures of exposure |  | NA |
|  |  | *Cross-sectional study—*Report numbers of outcome events or summary measures |  | NA |
| Main results | 16 | (*a*) Give unadjusted estimates and, if applicable, confounder-adjusted estimates and their precision (eg, 95% confidence interval). Make clear which confounders were adjusted for and why they were included | 7- 11 | Table 1, 2, 3 and 4 |
|  |  | (*b*) Report category boundaries when continuous variables were categorized |  | NA |
|  |  | (*c*) If relevant, consider translating estimates of relative risk into absolute risk for a meaningful time period |  | NA |

Continued on next page

| Other analyses | 17 | Report other analyses done—eg analyses of subgroups and interactions, and sensitivity analyses | 7 – 11 | Table 1, 2, 3 and 4 |
| --- | --- | --- | --- | --- |
| Discussion | | | | |
| Key results | 18 | Summarise key results with reference to study objectives | 11 - 12 | In the current study, sound environmental stimuli (Silence, Music, Noise) did not influence kinematics and neuromuscular activity during simple and challenging tasks in young healthy subjects, despite silence and music induced positive emotional responses, differently by noise which elicited opposite responses. Finally, the comparison between challenging and simple tasks showed some kinematic and electromyographic differences. |
| Limitations | 19 | Discuss limitations of the study, taking into account sources of potential bias or imprecision. Discuss both direction and magnitude of any potential bias | 13 | First, we cannot generalize our findings to all kind of music stimuli, because different genres of music including different rhythm contents and melodies more familiar with participants’ preferences could have produced different results in terms of motor performance. A second limitation is the lack of neurophysiological measurements or neuroimaging data to support the current findings. It may be interesting to assess if enriched environments including auditory stimuli modify brain activity in order to control potential distractors during execution of the same task.^35^ A third limitation may be the lack of control of cognitive involvement during the dual-task execution, for example counting the mistakes during the different auditory stimuli. Finally, the need to test the challenging task after the simple task might have masked potential differences between these two conditions. |
| Interpretation | 20 | Give a cautious overall interpretation of results considering objectives, limitations, multiplicity of analyses, results from similar studies, and other relevant evidence | 12 - 13 | The study of potential effects of sound environments on motor performance may play an important role when considering working environments, as noise is considered as one of the most dangerous workplace exposures.^40^ Several studies reported hearing impairments and cardiovascular disorders as consequence of a long-term noise exposure.^2^ Furthermore, noise exposure may adversely affect cognitive task performance, increasing the number of errors.^3^ To date, no studies have explored the noise effects on upper limb coordination. Although in our study music and noise exposition does not alter upper limb motor coordination, it is worth noting that the proposed experimental setting does not reproduce working characteristics, and it is not possible to exclude that results would be different for longer noise exposures. |
| Generalisability | 21 | Discuss the generalisability (external validity) of the study results | 12 - 13 | The study of potential effects of sound environments on motor performance may play an important role when considering working environments, as noise is considered as one of the most dangerous workplace exposures.^40^ Although in our study music and noise exposition does not alter upper limb motor coordination, it is worth noting that the proposed experimental setting does not reproduce working characteristics, and it is not possible to exclude that results would be different for longer noise exposures. |
| Other information | |  | | |
| Funding | 22 | Give the source of funding and the role of the funders for the present study and, if applicable, for the original study on which the present article is based | 13 | This research did not receive any specific grant from funding agencies in the public, commercial, or not-for-profit sectors. |

*Give information separately for cases and controls in case-control studies and, if applicable, for exposed and unexposed groups in cohort and cross-sectional studies.

**Note:** An Explanation and Elaboration article discusses each checklist item and gives methodological background and published examples of transparent reporting. The STROBE checklist is best used in conjunction with this article (freely available on the Web sites of PLoS Medicine at http://www.plosmedicine.org/, Annals of Internal Medicine at http://www.annals.org/, and Epidemiology at http://www.epidem.com/). Information on the STROBE Initiative is available at www.strobe-statement.org.
